# *Mycobacterium tuberculosis* in Wild Asian Elephants, Southern India

**DOI:** 10.3201/eid2303.161741

**Published:** 2017-03

**Authors:** Arun Zachariah, Jeganathan Pandiyan, G.K. Madhavilatha, Sathish Mundayoor, Bathrachalam Chandramohan, P.K. Sajesh, Sam Santhosh, Susan K. Mikota

**Affiliations:** Kerala Veterinary and Animal Sciences University, Kerala, India (A. Zachariah);; AVC College, Tamil Nadu, India (J. Pandiyan);; Rajiv Gandhi Centre for Biotechnology, Kerala (G.K.Madhavilatha, S. Mundayoor);; Scigenom Research Foundation, Kerala (B. Chandramohan, P.K. Sajesh, S. Santhosh);; Elephant Care International, Hohenwald, Tennessee, USA (S.K. Mikota)

**Keywords:** tuberculosis, *Mycobacterium tuberculosis*, elephant, *Elephas maximus*, India, zoonoses, tuberculosis and other mycobacteria, PCR, genetic sequencing, bacteria

## Abstract

We tested 3 ild Asian elephants (*Elephas maximus*) in southern India and confirmed infection in 3 animals with *Mycobacterium tuberculosis,* an obligate human pathogen, by PCR and genetic sequencing. Our results indicate that tuberculosis may be spilling over from humans (reverse zoonosis) and emerging in wild elephants.

Infection with *Mycobacterium tuberculosis* in domestic and wild animals of various species living in close contact with humans has been reported (*1*). Elephants in captivity are known to be susceptible to infection with *M. tuberculosis*, and there is a potential for transmission of *M. tuberculosis* between humans and elephants (*2**–**4*). In 2013, a case of tuberculosis (TB) in a wild elephant in Africa, which had been under human care, was reported (*5*), after which another case in a wild Asian elephant in Sri Lanka was reported (*6*). Habitat encroachment and competition for resources brings wild elephants into closer contact with humans, providing opportunities for zoonoses and reverse zoonoses to occur and for a previously unknown pathogen to emerge in captive free-ranging and wild elephant populations.

## The Study

**Figure 1:**
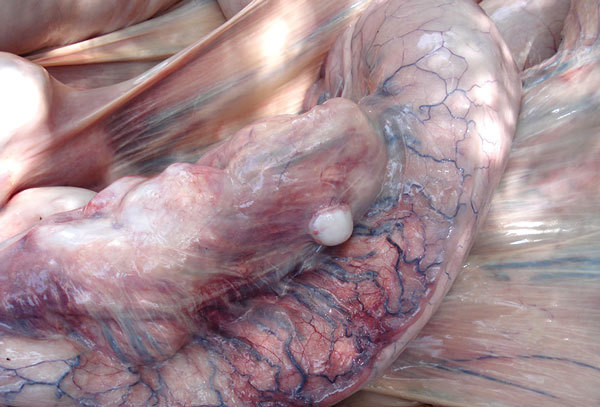
Intestine from a wild bull elephant, estimated at 20 years of age, Wayanad Wildlife Sanctuary, India, 2007. Multiple white-to-tan discrete nodules (granulomas) are protruding from the serosal surface, and less well-defined areas of pale discoloration are visible within the intestinal wall. Serosal blood vessels are markedly dilated, tortuous, and congested.

In March 2007, an emaciated wild bull elephant, estimated to be 20 years of age, died shortly after it was found recumbent in the Muthanga range of the Wayanad Wildlife Sanctuary in southern India (case 1). Postmortem examination revealed purulent exudates throughout the lungs, an enlarged liver, enlarged mesenteric lymph nodes, surface nodules containing caseated yellowish-white material (Figure 1). We found serosanguinous fluid in the pericardial sac and slightly hypertrophied heart ventricles. We saw focal areas of necrosis in the renal cortices but noted no other gross lesions. Ziehl-Neelsen staining of lung, liver, kidney, and mesenteric lymph node impression smears revealed numerous acid-fast bacilli. We confirmed the presence of *M. tuberculosis* by using PCR amplification of the targeted bacterial genome, gel documentation of the amplified products, and sequencing.

**Figure 2:**
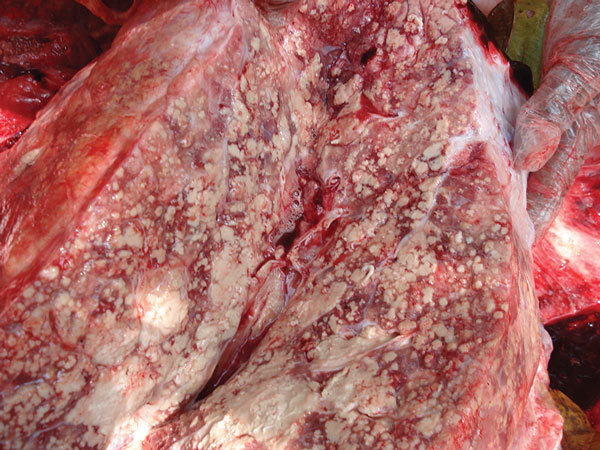
Lung from a bull elephant, estimated at 30 years of age, Kurichiyat Range, India, 2010. Note the multifocal to coalescing pale tan-to-white firm nodules (granulomas) effacing much of the lung parenchyma. Some areas of white chalky mineralization are also present.

Subsequently, a surveillance program was initiated (until March 2014), and all fresh elephant carcasses in the study area were examined for evidence of TB (n = 88). In May 2010, a bull elephant, ≈30 years of age, was found dead in the Kurichiyat range (case 2). Postmortem examination revealed extensive caseated lesions in the lungs (Figure 2) and mild mesenteric lymph node hypertrophy. In May 2013, TB infection was diagnosed in a bull ≈40 years of age that was found in the same forest range and had extensive caseated lung lesions (case 3). Both bulls were emaciated.

We fixed samples for histopathological studies in 10% formol saline and embedded them in paraffin. We found numerous acid-fast organisms in lung impression smears and tissue sections. Granulomatous lesions encapsulated by connective tissue with aggregated macrophages and central areas of necrosis were seen during histopathologic examination of the lungs for all 3 cases and of the kidney and liver in case 1. Langerhans-type giant cells were observed in cases 2 and 3 but not in case 1.

Tissues for molecular studies were collected in absolute alcohol. We extracted total DNA from tissues by using DNeasy Blood & Tissue Kit (QIAGEN GmbH, Hilden, Germany) according to the manufacturer’s protocol. DNA was subjected to a tetraplex PCR to differentiate between *M. tuberculosis* complex and nontuberculous mycobacteria. DNA was subjected to amplification and sequencing of the 3 target regions separately, 16S–23S internal transcribed spacer region, hsp65, and rpoB separately (*7*). *M. tuberculosis* H37Rv and *M. bovis* bacilli Calmette-Guérin genomic DNA was used as control DNA for the PCR studies.

We observed the expected 4-band pattern after tetraplex PCR. As the MTP40 fragment was amplified, *M. bovis* was ruled out because the *plcA* gene (mtp40), one of the members of the *plc* family of genes that code for the phospholipase C enzyme, is deleted in the *M. bovis* and *M. bovis* bacilli Calmette-Guérin RD5 region (*8*). Sequences that were generated were assembled and edited by using the alignment software Seqscape (http://www.seqscape.software.informer.com). BLAST (http://www.ncbi.nlm.nih.gov/BLAST/Blast.cgi) analysis of the edited sequences revealed that the elephant sequences showed 100% similarity with the *M. tuberculosis* genome fragment. We also used DNA for large sequence polymorphism analysis to determine the lineage of *M. tuberculosis* using RD239 and RD750 primers (*9**,**10*). The genomic deletion analysis revealed a deletion in RD239, which is characteristic of the Indo-Oceanic lineage (*10*), also referred to as the East African–Indian lineage (*11*).

## Conclusions

There are reports of mycobacterial infections in captive elephants in India from as early as 1925 (*12*). We report *M. tuberculosis* infection in wild elephants in India. In this study, 3 (3.4%) of 88 elephants undergoing postmortem examination were confirmed to be infected with *M. tuberculosis*. All 3 animals were emaciated, and we considered TB to be the cause of death.

The close interaction between humans and captive elephants is presumed to be a key risk factor for the interspecies transmission of TB. The epidemiology of TB among wild elephants, now documented in 3 countries, has yet to be elucidated. In our study, there were no known captive elephant releases or reintroductions into the study area, and the interaction between captive and wild elephants is considered negligible. However, native tribes do live within the park; many tribal members are employed by the forest department for protection and ecotourism activities. Tourists may visit specified areas only under supervision; there are no overnight facilities. Human–elephant conflict is a problem; most conflicts are caused by resident bulls. All 3 TB cases reported here were in bulls. Exposure of bulls to humans infected with TB during conflict activities is a possible explanation.

More than 3,000 native cattle reside within the sanctuary, cared for by the Animal Husbandry Department, Kerala State. No cases of TB among cattle have been reported. Cattle would be more likely to be infected with *M. bovis* than with *M. tuberculosis*, but comprehensive testing would be informative. Cattle living in close proximity to TB-infected humans can become infected with *M. tuberculosis* (*13*). Whether such infected cattle could then transmit *M. tuberculosis* to elephants through contamination of shared grazing lands is yet another research question.

The *M. tuberculosis* complex is thought to have emerged as a human pathogen in Africa rather than arising from an animal source (*14*). Although the epidemiology has not been defined, our study and previous reports indicate that *M. tuberculosis* appears to be spilling over into elephants (reverse zoonosis) and emerging among wild elephant populations. Although these cases may have resulted from individual introductions, if *M. tuberculosis* becomes established, wild elephants and other susceptible species will be at risk.

Ecologic, environmental, or demographic factors that place animals or humans at increased contact can contribute to disease emergence. Certainly, the increased human–elephant conflict in India and other Asian elephant range countries attests to the narrowing interface between humans and elephants. This study suggests that *M. tuberculosis* is emerging in the largest single population of Asian elephants in India. Continued surveillance in India and other Asian elephant range countries is warranted.
